# Beyond VEGF and TGF-β: A Comprehensive Review of Growth Factor Pathways in the Pathophysiology of Uterine Leiomyomas

**DOI:** 10.3390/biology15010092

**Published:** 2026-01-01

**Authors:** Nuray Rozmurat, Sanja Terzic, Peng Zhao, Gauri Bapayeva, Kuralay Kongrtay, Matthew Naanlep Tanko, Milan Terzic

**Affiliations:** 1 School of Medicine, Nazarbayev University, Zhanybek-Kerey Khans Street 5/1, Astana 010000, Kazakhstan; 2 Department of Medicine, School of Medicine, Nazarbayev University, Zhanybek-Kerey Khans Street 5/1, Astana 010000, Kazakhstan; sanja.terzic@nu.edu.kz (S.T.); matthew.tanko@nu.edu.kz (M.N.T.); 3 Department of Obstetrics, Women’s Hospital, Zhejiang University School of Medicine, No.1 Xueshi Road, Hangzhou 310006, China; zhaopeng0622@zju.edu.cn; 4 Clinical Academic Department of Women’s Health, CF “University Medical Center”, Turan Ave. 32, Astana 010000, Kazakhstan; gauri.bapaeva@umc.org.kz (G.B.); kkongrtay@nu.edu.kz (K.K.); milan.terzic@nu.edu.kz (M.T.); 5 Department of Surgery, School of Medicine, Nazarbayev University, Zhanybek-Kerey Khans Street 5/1, Astana 010000, Kazakhstan

**Keywords:** uterine leiomyoma, VEGF, TGF-β3, angiogenesis, extracellular matrix, growth factors, clinical management

## Abstract

Uterine leiomyomas or fibroids, are non-cancerous smooth muscle proliferations of the uterus, occurring mostly in women of reproductive age. Their pathogenesis involves complex growth factor interactions that regulate cellular proliferation, extracellular matrix (ECM) remodeling, and angiogenesis in myometrium. Women affected by fibroids often have a range of consequences such as infertility, endometriosis, and dysmenorrhea. Several growth factors such as vascular endothelial growth factor (VEGF), transforming growth factor (TGF-β), and platelet-derived growth factors (PDGF) have long been described as key regulators of angiogenic and fibrotic activities in fibroid tissue. Moreover, we summarized updated information between 2015 and 2025 following strictly inclusion/exclusion criteria and key research areas, including growth factors and its isoform-interaction, their roles within key signaling pathways, and the contribution of ECM deposition in uterine fibroids development and growth. Implementing growth factors in the clinical research field can develop new biomarkers and treatment options, focusing on effective and advanced management of uterine fibroids.

## Simple Summary

Uterine fibroids, sometimes known as uterine leiomyomas, are characterized as a non-cancerous gynecological disorder that can develop in the wall of the uterus, mainly in women who are able to become pregnant. They cause heavy bleeding, anemia, pain, and sometimes fertility issues, which represent a significant adverse impact on women’s health. Certain natural substances in the body, known as growth factors, send out specific chemical messages, which can push fibroids to develop, supporting fibroid cell multiply; create new blood vessels; and produce extra tissues. The main function of the growth factor is to support tissue repair and healing the injuries; however, in the case of fibroids, they become overly active, leading to the excessive development and growth of fibroid tissue. This review highlights the importance of growth factors in uterine fibroid biology, analyzing and searching scientific findings from the last 10 years. It explains how scientists have learned how growth factors work together and their influence on the fibroid development and structure. Additionally, recent scientific advances, including biomarkers and treatment options, can be more effective and invasive.

## 1. Introduction

Uterine fibroids (UFs), also known as leiomyomas, represent the most prevalent tumors among women and individuals assigned female at birth [1]. They are benign tumors consisting of disorganized myofibroblasts embedded within significant amounts of extracellular matrix, which constitute a considerable portion of the tumor volume [2]. It is observed that approximately three-fifths of women during their reproductive age experience UFs [3,4]. The reported incidence of uterine leiomyomas may not be accurate, since they are asymptomatic, and consequently underestimate the real rates in clinical practice [5]. Several symptoms exist in women with UFs: a heavy and irregular menstrual cycle, dysmenorrhea, pelvic pressure, and infertility [6,7,8]. Fibroids originate from myometrial smooth muscle cells, whereas fibroid development occurs under the influence of hormonal factors, growth factors, and extracellular matrix accumulation [1].

Epidemiology research findings reveal several factors which lead to leiomyoma growth and development; furthermore, risk factors including hormonal, genetic, and environmental factors significantly play an essential role in the etiology of fibroids. The pathogenesis of UFs is regulated by risk factors that trigger chronic inflammation, DNA damage, and genetic mutations [5]. Researchers identified that insufficient consumption of fruit and green vegetables, inadequate vitamin D levels, and ingestion of contaminated food collectively promote the development of uterine leiomyomas [9,10,11,12,13], consequently, women who suffer from obesity or with excess body weight are linked with a slightly higher risk of developing uterine fibroids [14,15,16,17]. As heavy metals have a non-biodegradability structure, they enter human bodies through contaminated food. For example, studies have shown that mercury (Hg), a toxic heavy metal primarily found in fish, occurs at higher concentrations in women with symptoms of uterine fibroids [16].

The premenopausal phase and ages above 40 play another important role in the development of fibroids [5], also myomas develop only after puberty occurs [17].

**Figure 1:**
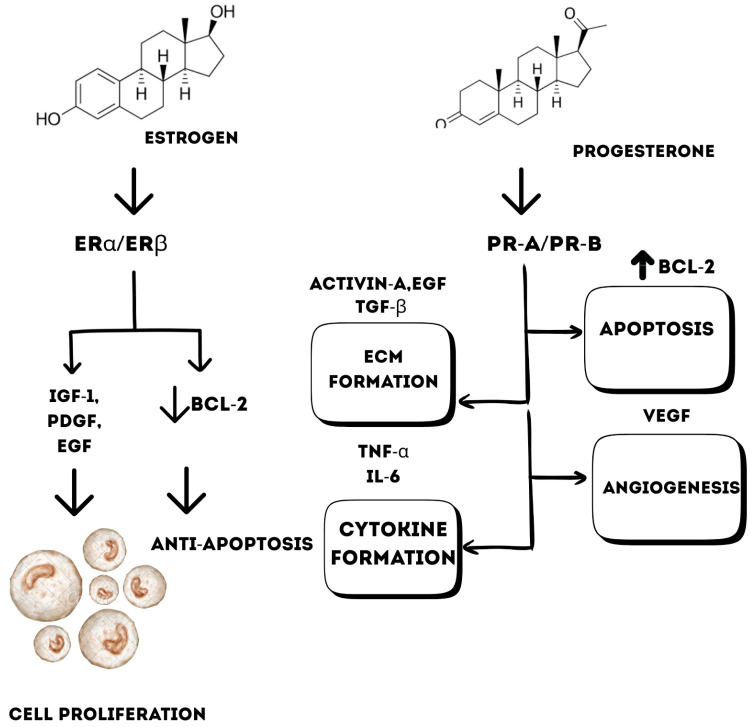
Progesterone and estrogen signaling in uterine leiomyoma pathogenesis.

The cell development is associated with ovarian hormones, estrogen, and progesterone [18]. Also, steroid hormones and growth factors contribute significantly to fibroid growth and development [19]. Estrogen binds to its receptors, ERα and ERβ, and promotes cell proliferation by upregulating growth factors such as the insulin-like growth factor 1 (IGF-1), epidermal growth factor (EGF), and platelet-derived growth factor (PDGF) [20]. Whereas through mitogen-activated protein kinase (MAPK) pathways, estrogen inhibits bcl-2 expression, thereby leading to anti-apoptosis [21], while progesterone promotes bcl-2 leading to inhibition of fibroid cell apoptosis [20]. Progesterone regulates cytokine production, such as tumor necrosis factor (TNF-α) and interleukin 6 (IL-6) [22]; enhances angiogenesis through vascular endothelial growth factor (VEGF); and promotes extracellular matrix deposition by activating Activin A [22], transforming the growth factor beta (TGF-β), and EGF pathways in leiomyoma tissue [20] (Figure 1).

In view of recent findings about the influence of growth factors in uterine fibroids, this review aims to evaluate and provide a brief overview of VEGF and the transforming growth factor beta 3 (TGF-β3) in the fibroid pathogenesis, the role of other growth factors, risk factors, and how growth factor interactions with isoforms influence disease progression. Further findings will provide brief insights into the interaction between growth factors and leiomyomas, and the identification of effective treatment options could lead to improved clinical management and developed new therapeutic biomarkers for uterine leiomyomas.

## 2. Materials and Methods

The narrative review follows the Preferred Reporting Items for Systematic Reviews and Meta-Analysis guidelines (PRISMA). The narrative review was chosen for this study as it offers a general, qualitative synthesis of the recent literature such that authors may study complex or new topics in significant detail. It is different from systematic reviews, which have no flexibility in combining and matching study designs, historical environments, and specialist opinion. It allows the detection of gaps in research, theoretical methodologies, and emerging scientific directions that are not yet amenable to meta-analysis. Narrative reviews are especially useful for condensing wide fields, placing new results into context, and forming hypotheses for subsequent studies.

### 2.1. Search Strategy

Because of the large amount of uterine fibroid studies and the duplication of records, the reviewers agreed to use only Scopus and Pubmed. The search was performed between 1 January 2015 and 30 September 2025. The search strategy MeSH terms (if available) and the following free text words were included: “uterine fibroid”, “leiomyoma”, and “uterine leiomyoma” (MeSH Unique ID: D007889); “platelet-derived growth factor”, “PDGF”, “PDGF-A”, “PDGF-B”, “PDGF-C”, and “PDGF-D” (MeSH Unique ID: D017479); “transforming growth factor beta”, “TGF-β”, and “TGF-beta” (MeSH Unique ID: D016212); “vascular endothelial growth factor” and “VEGF” (MeSH Unique ID: D042442); “fibroblast growth factor” (MeSH Unique ID: D005347); “tumor necrosis factor-alpha” or “TNF-α” (MeSH Unique ID: D014409); “epidermal growth factor” (MeSH Unique ID: D004815); and “human”, “human tissue”, or “clinical” (MeSH Unique ID: D006801).

### 2.2. Eligibility Criteria

**Figure 2:**
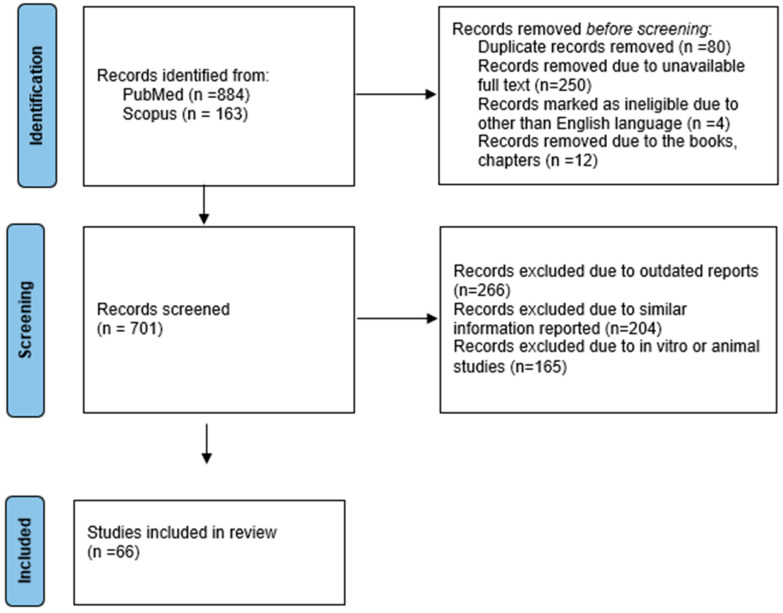
Data collection flowchart (using Prisma guideline).

The following inclusion criteria were applied: articles published only in English between 2015 and 2025, human-based studies including serum, tissue, clinical analysis related to uterine fibroids, articles that summarized evidence about at least one growth factor receptors, or PDGF, TGF-β3, VEGF, and growth factor functions such as proliferation, extracellular matrix (ECM) deposition, or angiogenesis. The following exclusion criteria were applied: experimental (animal and in vitro) studies, articles published in languages other than English, and articles published before 2015 (Figure 2).

## 3. Results

### 3.1. Vascular Endothelial Growth Factor (VEGF)

Vascular endothelial growth factor governs angiogenesis, which is critical for neoplasm development and growth [2], as well as for microvascular permeability, and is expressed from several tumor cells [23]. Fibroids require high levels of angiogenesis to support their growth and development. As fibroid size and volume increase, low-oxygen areas develop, which activate VEGF-A to promote new blood vessel formation. The core functions of VEGF include cell proliferation, migration, and vascular endothelial cell development [23]. The expression of VEGF-A is higher in leiomyomas compared to peripheral myometrium; in addition, VEGF, mRNA, and protein are consistently observed in fibroid tissue [24]. Compared to other growth factors, VEGF and TGF-β reveal significantly higher levels in leiomyoma rather than normal myometrium [2]. In younger and perimenopausal women, higher VEGF-A levels are associated with large fibroids, consequently, expression intensity is affected by patient age, fibroid size, and hormonal status [25]. Vascular endothelial growth factor receptor (VEGFR) subtypes such as VEGFR-1 and VEGFR-2 are detected in myometrial smooth muscle cells and fibroids, highlighting their key role in fibroid biology [24]. While VEGFR-2 through PLCγ-PKC-Raf-MEK-MAPK signaling pathway sends signals via VEGF to the nucleus to activate DNA synthesis and manage endothelial cell proliferation [26].

VEGF-A contains many isoforms, namely VEGF-A111, 121, 145, 165, 183, 189, and 206, produced by alternative splicing of exons 6A, 6B, 7A, and 7B. VEGF promotes endothelial cell proliferation and activates subsequent signaling pathways, including additional growth factors [27,28]. VEGF-A activates VEGFR-2 on endothelial cells, facilitating Tyr1214 phosphorylation and initiating angiogenic signaling for the growth of new vessels [29]. Isoforms vary in length, extracellular matrix (ECM) affinity, and permeability [30]. The extracellular matrix plays a major role in fibroid development, acting as a major source for growth factors, cytokines, chemokines, and proteases, influencing cell proliferation, differentiation, and matrix modification [29]. VEGF-A145, VEGF-A189, and VEGF-A206 demonstrate significant binding to the extracellular matrix and cell surfaces, while VEGF-A111 and VEGF-A121, which lack exons 6 and 7, display more infusibility [31]. VEGF-A165 is the predominant and most effective isoform in stimulating angiogenesis [32]. The VEGF-A121 isoform forms narrow, highly permeable vessels with reduced angiogenic complexity, highlighting its lack of extracellular matrix binding, whereas the VEGF-A189 isoform binds with high affinity to the extracellular matrix and stimulates irregular vascular branching. These isoform-specific variations produce several vascular structures and phenotypes, leading to the mitogenic and non-mitogenic pathways in fibroid development [29]. Although VEGF-A165 has been well investigated in relation to uterine fibroids, the functions of VEGF-B, VEGF-C, and VEGF-D are yet insufficiently investigated. Additional study is needed to determine their role in fibroid angiogenesis and proliferation [32]. Clinical evidence showed that VEGF-A expression was higher in both small and large myomas, highlighting that angiogenesis is independent of myoma size and tumor development [18].

Tumor angiogenesis and vascular remodeling occur through the contribution of VEGF, FGF-2, and PDGF growth factors. Also, FGF-2 directly stimulates angiogenic effects and endothelial proliferation, while mainly VEGF downregulates endothelial migration [33].

The VEGF, IGF-1, and TGF-β markers can be key biomarkers related to fibroid development and shrinkage after UAE treatment [16,17,18,19,20,21,22,23,24,25,26,27,28,29,30,31,32,33,34]. Mifepristone drug therapy has been stated to downregulate VEGF expression in leiomyoma cells, which consequently decreases cell survival. However, targeting VEGF interventions can display therapeutic potential in preclinical evaluations of uterine fibroids [2]. Most of the research focused on VEGF-A, while the function of isoforms and VEGF-B, VEGF-C, and VEGF-D in fibroid biology are still unclear. Further investigations are required to define growth factor signaling as a technique for controlling fibroid development through limiting vascular supplies. Clinical evidence is still unclear; nevertheless, researchers believe that anti-angiogenic factors will help identify specific anti-angiogenic treatment [32].

### 3.2. Transforming Growth Factor-β (TGF-β)

Transforming growth factor-β (TGF-β) is recognized as a pivotal cytokine associated with the myometrium, among others implicated in the biology of uterine fibroids (UFs). TGF-β functions as a key cytokine that regulates human cell proliferation and differentiation to cause fibrotic diseases that affect myocarditis, nephropathy, and inflammatory bowel disease. The three TGF-β isoforms TGF-β1, TGF-β2, and TGF-β3 activate their receptors through TGF-βR-I and TGF-βR-II [19]. The TGF-β family regulates inflammation, cell cycle progression, cellular proliferation through paracrine, and autocrine signaling pathways. The TGF-β cytokine family includes several functions: guiding macrophages and fibroid cells, acting as a chemotherapy trigger, restricting cell development in specific cell types, inducing programmed cell death, and managing ECM formation and development [19].

Leiomyoma cells produce excessive amounts of ECM components, including collagens and fibronectin and proteoglycans, when compared to normal myometrial cells [35]. The ECM maintains equilibrium through TGF-β3, which stimulates the formation of fibronectin and collagens and changes the expression of matrix metalloproteinases (MMP) members [36], but simultaneously blocks the genes that cause ECM degradation. Also, TGF-β signaling acts together with VEGF to regulate excessive ECM accumulation [37]. Excessive ECM cause abnormal bleeding, pelvic pain, and stiffness [5]. GF-β3 and Activin A stimulate increased mRNA levels in collagen and proteoglycans [36].

TGF-β signaling is regulated by multiple feedback mechanisms that function at the receptor level and both upstream and downstream of the receptors. Studies indicated that progesterone promotes TGF-β and Smad signaling pathways, which lead to stimulation of proliferation and growth [38]. The feedback mechanisms at the receptor level involve TGF-β-induced expression of inhibitory Smad7. This protein forms a complex with the ubiquitin ligase Smurf and the PP2C phosphatase. Smad7 binds to transforming growth factor-β receptor type I (TbRI), which creates a path for Smurf and protein phosphatase 2C (PP2C) to reach the receptors, leading to their receptor binding, degradation, dephosphorylation, and deactivation [39]. The SMAD protein complex serves as a TGF-β signaling pathway to control cell growth and survival and fibrosis development through its ability to regulate ECM deposition and remodeling processes [28]. Also, through Smad and non-Smad signaling pathways TGF-β suppresses ECM components collagen I and III in leiomyoma cells [20].

Vitamin D3 produces therapeutic effects for uterine leiomyomas through its dual action of controlling TGF-β-responsive genes and its blocking effect on Wnt/β-catenin and mammalian target of rapamycin (mTOR) signaling pathways. Research shows Vitamin D3 stops TGF-β3 from activating Smad2 phosphorylation and prevents Smad2 and Smad3 from entering the nucleus in leiomyoma cells [40]. The Wnt/β-catenin pathway functions as a primary regulatory mechanism that directs TGF-β to produce extracellular matrix in smooth muscle tissues. Research has not fully determined the exact function of Wnt/β-catenin signaling during leiomyoma formation and its relationship with extracellular matrix remodeling factors [41].

### 3.3. Fibroblast Growth Factors (FGFs)

The main functions of fibroblast growth factor are cell migration, differentiation, angiogenesis (in specific medicine fields), wound repair, neurogenesis, and intracellular signaling [42]. It is divided into two major categories. Acidic FGF (aFGF or FGF-1) shows higher expression in leiomyomas compared with the surrounding myometrium [18], while basic FGF (bFGF or FGF-2) is excreted mainly by smooth muscle cells (SMCs) and macrophages [42]. Both FGFs serve as potent mitogens, promoting smooth muscle cell and fibroblast proliferation. However, bFGF has a significant influence on the angiogenesis process, it binds to FGFR1 on endothelial cells, thereby triggering receptor dimerization and phosphorylation, leading to the activation of signaling cascades that mediate motility, proliferation, and survival [43]. Basic FGF, which is secreted by tumor cells, supports the tumor microenvironment and acts as a regulator of several transduction signals. Also, FGFR1–4, one of the major receptors of FGFs, is observed mainly in endothelial cells (ECs) and SMCs, with increased expression frequently observed in fibroid tissue [42]. The heparin-binding FGFs are present in myometrium and leiomyoma tissue [20].

Through MAPK and PI3K/AKT pathway activation bFGF binds to FGFR1, which causes dimerization and internalization of the receptor-ligand complex. Thereby, this activity promotes fibroid proliferative and angiogenic signaling [43]. The main cytogenetic alteration in uterine fibroids results from translocation events, which have been associated with increased tumor size. This alteration is believed to influence fibroid growth. Research shows that this factor triggers bFGF mRNA production through signaling pathways that lead to tumor growth and development [24,25,26,27,28,29,30,31,32,33,34,35,36,37,38,39,40,41,42,43,44].

The clinical implications of uterine fibroids remain unclear, but FGFs promote cell proliferation, angiogenesis, and extracellular matrix remodeling. Also, FGF together with VEGF acts as a strong angiogenesis driver, initiating endothelial cell proliferation and migration [45]. Fewer therapeutic managements have focused on FGFs, compared with VEGF and TGF-β. However, its key role in the fibroid biology describes them as potential candidates for future interventions [42].

### 3.4. Platelet-Derived Growth Factor (PDGF)

Platelet-derived growth factor (PDGF) is initially identified as a granule constituent released autocrine way upon platelet activation [46], which plays an important role in regulating cell growth, proliferation, and differentiation [47]. PDGFs and their receptors, such as PDGFRα and PDGFRβ expression, have been observed in multiple cancer types including non-small cell lung carcinoma (NSCLC), gastrointestinal stromal tumors (GIST), pancreatic carcinoma, breast and ovarian carcinomas, hepatocellular carcinoma, and diverse neuroendocrine tumors [46].

PDGFs exist as four distinct monomeric polypeptide chains which include PDGF-A, PDGF-B, PDGF-C, and PDGF-D. The chains form five dimeric isoforms through disulfide bonds which include four homodimers (PDGF-AA, PDGF-BB, PDGF-CC, PDGF-DD) and one heterodimer (PDGF-AB). PDGF isoforms develop through two separate mechanisms. The PDGF-AA, PDGF-AB, and PDGF-BB dimers need intracellular breakdown for activation before they start their secretion process while PDGF-CC and PDGF-DD need external activation after their release as inactive forms [46]. Also, PDGF-AA, PDGF-BB, and PDGF-CC and their receptors have been seen mostly in leiomyoma rather than normal cells [32].

PDGF functions as an essential promoter of smooth muscle cell proliferation, extracellular matrix formation and secretion. Research has shown that the amount of PDGF is greater in uterine fibroids rather than in adjacent myometrium: around 80% of upregulation is identified [44]. In vivo experiments revealed that PDGF-C extends the survival of fibroid-derived smooth muscle cells in Matrigel plug models, displaying its function in sustaining cell viability [20]. Also, it stimulates DNA and protein synthesis and cell proliferation in myoma cells, as well as activating collagen a1, PCNA production, and the MAPK pathway [24,25,26,27,28,29,30,31,32,33,34,35,36,37,38,39,40,41,42,43,44]. The binding of PDGF-BB and PDGF-DD to PDGFRβ on the cell surface causes activation of the JAK/STAT pathway, while interaction between PDGF and PDGFR promotes PI3K/AKT, Ras/MAPK, PLC-γ, and JAK/STAT signaling pathways. Therefore, it leads to the regulation of key processes such as proliferation, survival, and functional adaptation of the cell, initiating transcriptional alterations [48].

PDGF expression increases, downregulates activin and myostatin levels under steroid hormone estrogen influence, and encourages myoma cell proliferation by growth factor and signaling regulation [44].

In vitro, PDGF-BB is a key active compound for cell cultures, facilitating mitogenesis and chemotaxis of periodontal ligament cells more efficiently than PDGF-AA and PDGF-AB [49]. According to the Navarro et al. (2021), women with uterine fibroids have increased level of angiogenic activity, PDGF interacts with transforming growth factor-β (TGF-β), an important fibrogenic cytokine, to promote tissue remodeling and fibrosis [28]. The interaction between PDGF and TGF-β3 pathways has been identified as a regulatory complex network in fibroid development control [50].

### 3.5. Insulin-like Growth Factors (IGFs)

IGF-1 activates protein synthesis and proliferation, encourages progressive growth, contributes to the improvement of the nervous system, protects it from apoptosis, and supports stem cells [51]. IGF-1 through IGF-1R manages myoblast proliferation. IGF-1 also promotes ECM components such as collagen and proteoglycans [52]. IGF-1 is one of the important factors in fibroid growth, because it is much higher than other growth factors in myometrium and leiomyoma tissues. However, there is no difference in mRNA IGF-1 expression in both tissues. Endocrine, autocrine, and paracrine stimulation of IGF-IR, regulated by IGF-1 and IGF-2, plays an important role in fibroid development and shows high levels in both tissues [53]. IGF-1 signaling includes many intracellular pathways, such as the Ras/Raf/MAPK and the PI3K pathways. The activation of PI3K is regulated by the RTK transduction pathway, which controls cell proliferation and sends extracellular signals within the cell. Key hormonal regulator estrogen contributes to an increase in the IGF-1 gene in leiomyoma tissue [53].

IGF-2 and its receptor initiate TGF-β pathway, leading to abundant ECM accumulation and triggering differentiation of fibroblasts into myofibroblasts, thereby it facilitates fibrosis development and growth. IGF-2 is a part of the insulin family, which mainly facilitates cell proliferation and migration process. It establishes a fibrotic environment which can facilitate fibrotic disease development and progression [54]. Studies found that during the laboratory experiment IGF2 increases the level of proliferation in uterine fibroid cells and promotes the ERK pathway [55]. According to research evidence, IGF-1 together with VEGF can be used as prognostic biomarkers to evaluate patients’ condition with uterine fibroids after UAE. Because the low serum level of IGF-1 and VEGF in patients after UAE treatment demonstrated significant improvement in progression-free survival [34].

IGF-binding proteins (IGFBP) bind IGFs with high affinity, the essential modulator of autocrine and paracrine activities of IGFs. Also, they suppress several IGF activities such as cell migration, proliferation, and differentiation. In addition, they play an essential role in fetal development and growth and placental function and regulation, by inhibiting IGF activities [56].

### 3.6. Pro-Inflammatory Cytokines (TNF-α, IL-6, IL-1β)

Tumor necrosis factor-α (TNF-α) is higher in fibroid tissue compared to normal myometrium [40], thereby increasing Activin A expression in myometrial and leiomyoma cells, highlighting its importance in extracellular matrix (ECM) synthesis [57]. The cytokine key functions also include regulating immune function, cell growth and differentiation, apoptosis, and inflammatory responses [37]. The expression of cytokine regulates two receptors, TNFR1 which controls cytotoxic, pro-inflammatory, and pro-apoptotic responses, and TNFR2 which supports the regulatory T cell stabilization [58]. Through ERK pathway, TNF-α promotes human leiomyoma smooth muscle cells migration and MMP-2 production [59].

Cytokines and chemokines drive fibroid development by stimulating excessive ECM production and promoting smooth muscle cell proliferation [60]. This pathogenic process is likely driven by the high expression of pro-inflammatory cytokines, as observed in uterine fibroids [39,40,41,42,43,44,45,46,47,48,49,50,51,52,53,54,55,56,57,58,59,60,61]. Hematopoietic growth factors (HGFs) are extracellular, glycosylated proteins [62], whose interaction with VEGF facilitates vascular development, highlighting its biomarker role in uterine fibroids. Inhibition of NF-κB pathway decreases TNF-α, IL-6, and IL-1β levels, which leads to a reduction in fibroid size [61].

### 3.7. Epidermal Growth Factor (EGF)

**Figure 3:**
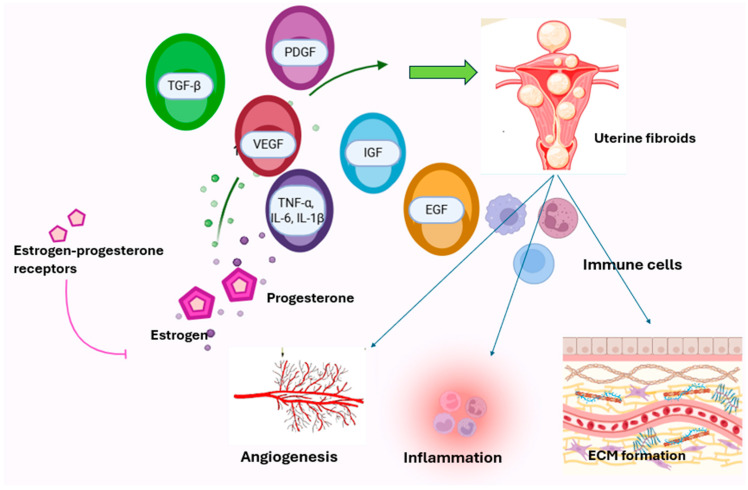
Integrated network of hormones, growth factors, and cytokines in uterine fibroid development. The figure shows how estrogen and progesterone activate uterine fibroid growth by stimulating their receptors and collaborating with growth factors such as TGF-β, VEGF, PDGF, IGF, EGF, and inflammatory cytokines (TNF-α, IL-6, IL-1β). Immune cells support uterine fibroid development; its pro-inflammatory microenvironment regulates tumor growth. Overall, growth factors and cytokines play an essential role in uterine fibroids by regulating angiogenesis, inflammation, and ECM formation.

Through extracellular signal-regulated kinase (ERK-MAPK), PI3K-AKT, SRC, PLC-γ1-PKC, JNK, and JAK-STAT pathways, EGF manages angiogenesis, cell death, proliferation, and migration [62]. The expression of EGF decreased, whereas EGFR expression increased by estrogen [21]. Also, EGF activates DNA synthesis in leiomyoma, however EGFR demonstrates equally in both leiomyoma and myometrial smooth muscle cells [20]. [Table 1 and Table 2] [Figure 3].

## 4. Discussion

This systematic review highlights that uterine fibroids are managed by growth factor complex networks that promote cellular proliferation, extracellular matrix stimulation, and angiogenesis [24]. Our study especially focuses on VEGF, PDGF, and TGF-β growth factors. Research demonstrated that women with UFs have an experience with abnormal vascular structure and several pathophysiology features caused by angiogenic growth factors such as VEGF and PDGF [28]. In addition, cytokines play an important role in UF symptoms; they are involved in several clinical outcomes, such as menstrual pain and reproductive dysfunction [63]. The inflammatory cytokine TNF-α promotes tissue fibrosis and is highly expressed in uterine fibroids [63].

The extracellular matrix is an essential storage system that supports and promotes growth factors and hormones. ECM components such as collagen, fibronectin, and versican experience increased levels and are regulated through growth factor signaling pathways. In addition, estrogen and progesterone act together in the activation of ECM components. These research findings reveal the strong relationship among the extracellular matrix, growth factors, their receptors, and subsequent signaling cascades in the pathogenesis of uterine fibroids [39].

Excessive ECM accumulation leads to uterine fibroids [12]. ECM consists of collagen, fibronectin, proteoglycans, MMPs and TIMPs, and LOX cross-linking enzymes. Mostly their role in UFs includes cell migration, differentiation, inflammation, and ECM remodeling, revealing high levels in leiomyoma. Growth factors such as TGF-β3, VEGF, PDGF, and FGF stimulate increasing mRNA levels in ECM components [19,36,39,64,65,66]. Another impact on angiogenesis was caused by ECM, where ECM components and their interaction with each other regulates how growth factors attach to the ECM [29]. Estrogen and progesterone regulate ECM components at different levels, either increasing, reducing, or modulating their expression.

Future research investigation may involve TGF-β3 and VEGF growth factor’s role in uterine artery treatment and determine PDGF isoforms’ role in the development of fibroids, highlighting the importance of ECM stimulation, hormonal factors, cell proliferation, and angiogenesis. Concerning the uterine artery embolization process, measuring TGF-β3 and VEGF blood sample levels and menstruation cycle and pain on the quality of life of patients will be another long-term outcome treatment [7,23]. Other methods will include spatial gene-expression mapping, single cell RNA sequence analysis, and multiplex protein detection analysis. These clinical and molecular level studies help to identify new diagnostic treatment options and biomarkers and navigate the development of patient-centered therapeutic strategies for women of reproductive age with uterine fibroids symptoms [Table 3].

### Strengths and Limitations

The critical systematic review has several strengths. It evaluates evidence from both biological and molecular pathway studies, providing in-depth analysis of the growth factor and its isoform pathways in uterine leiomyoma development. In addition, the recent peer reviewed literature from 2015 to 2025, eligibility criteria, structure, and highlighting gaps in the existing literature improve scientific reliability and quality of the analysis.

Despite its strengths, the systematic review has certain limitations. It is difficult to make conclusions based on a small cohort and limited large-scale human studies connecting PDGF isoforms, VEGF, and TGF-β3 in fibroid biology, with the absence of consistent outcome evaluation. Other limitations include significant differences in study design, sample size, and outcome results, which may contribute to publication bias.

VEGF and TGF-β play a central role in the diagnosis, treatment, and therapeutic experiments of uterine fibroid biology. One of the key roles of VEGF includes angiogenesis, vascular permeability, and fibroid development, in addition VEGF assessment could be a potential biomarker for disease development and uterine artery embolization treatment response. While TGF-β expression may serve as a key regulator of collagen deposition, ECM accumulation, and tissue remodeling, which can be an anti-fibrotic therapeutic agent. Growth factor measurements in tissue could help to determine patients’ disease stage and provide early accurate predictions of treatment outcomes. Progesterone-receptor modulating agents, considered as growth factor-targeted therapies, can help to decrease fibroid size and blood supply without affecting fertility. The integration of VEGF and TGF-β into clinical practice will support healthcare professionals to develop personalized management strategies for uterine fibroid patients.

## 5. Conclusions

The complex molecular interaction between TGF-β, VEGF, and PDGF isoforms has been the most intensively demonstrated as a key regulator of angiogenesis, cell growth and development, and extracellular matrix accumulation within fibroid biology. However, a feedback regulation mechanism and pathways integration under hormonal influence has not yet emerged. On the other hand, molecular and proteomic analyses and translational research on growth factors could lead to fibrosis regulation and development of tumors in myometrial tissue. We hypothesize that how specific growth factors and their pathway interact with each other will be important factors for future treatments. Previous studies have been focused on single growth factors, while we suggest isoform-specific binding and crosstalk between growth factors can be efficient and improve patient satisfaction and quality of life. Nevertheless, future studies on growth factors are needed to identify new therapeutic biomarkers, support patient-centered management strategies for women with uterine fibroid diagnosis.

## Figures and Tables

**Table 1:** Growth factors, angiogenic mediators, and cytokines implicated in uterine fibroid development and growth.

Marker (Isoforms/Receptors)	Expression Pattern in Fibroid vs. Myometrium	Principal Receptors and Downstream Pathways	Biological/Clinical Function(s) in Fibroid Pathogenesis	Crosstalk with Other Mediators	References
VEGF-A (VEGF121, VEGF165, VEGF189; VEGFR1/VEGFR2)	Most studies show increased total VEGF-A mRNA/protein in fibroids vs. matched myometrium; isoform-specific data limited but VEGF165 predominates and VEGF189 is the principal matrix-bound form	VEGFR2 → PLCγ/PKC, PI3K/AKT, MAPK; increased endothelial proliferation, permeability and angiogenesis	Cell proliferation, migration and vascular endothelial cell development and may influence growth after UAE	FGF2 and PDGF for angiogenesis; interacts with TGF-β signaling to promote ECM deposition	[23,26,29,33,37,45]
TGF-β family	TGF-β consistently high ↑ increase in leiomyomas vs. myometrium	SMAD2/3 and Wnt/β-catenin pathway	Activate increased in mRNA expression of collagen, proteoglycans	Cross-talk with VEGF, promoting ECM accumulation	[2,36,37,39,41]
PDGF (PDGF-AA/BB; PDGFRα/β)	PDGF expression increased in many fibroid tissues	PI3K/AKT, ERK/MAPK → proliferation and fibrosis	Downregulates activin and myostatin levels under steroid hormone estrogen influence, encourages myoma cell proliferation	Synergy with TGF-β pathways	[20,45,48,50]
FGF	Frequently increased in fibroids and surrounding stroma	FGFR1/2 → MAPK, PI3K → mitogenic, angiogenic effects	Angiogenesis; increases matrix deposition	Potentiates VEGF-driven angiogenesis	[20,42,43,45]
EGF/EGFR	EGFR is regulated equally in the leiomyoma and MSMC	EGFR → MAPK/PI3K signaling	Activate DNA synthesis in leiomyoma	Interacts with IGF and PR/ER pathways	[20,21,62]
IGF-1/IGF-2	High in uterine fibroids and myometrium than other growth factors	PI3K/AKT, MAPK	Excessive ECM accumulation and its components	Crosstalk with TGF-β, VEGF	[34,51,52,54]
Pro-inflammatory cytokines (TNF-α, IL-6, IL-1β)	Pro-inflammatory cytokines are highly expressed in uterine fibroids	NF-κB, ERK activation	Extracellular matrix (ECM) synthesis, apoptosis	Potentiates TGF-β and VEGF signaling	[27,39,61]

**Table 2:** Growth factor and cytokines signaling pathway.

Growth Factor/Cytokine	Key Signaling Pathway	Role of Signaling Pathways in Fibroid Biology	References
VEGF (VEGF-A, B, C, D)	PLCγ–PKC–Raf–MEK–MAPK; PI3K/AKT	MAPK promotes endothelial proliferation; PI3K/AKT supports endothelial survival and permeability	[26]
TGF-β family	SMAD2/3–SMAD4; MAPK; PI3K/AKT	SMAD signaling activates fibrosis and ECM deposition; non-SMAD pathways support proliferation and angiogenic crosstalk	[28,38]
FGFs (FGF-1, FGF-2)	MAPK; PI3K/AKT	MAPK mediates smooth muscle and fibroblast proliferation; PI3K/AKT promotes angiogenic survival signaling	[43]
PDGF (AA, BB, CC, DD)	PI3K/AKT; Ras/MAPK; PLC-γ; JAK/STAT	PI3K/AKT supports stromal cell survival; MAPK stimulates proliferation; JAK/STAT regulates fibrotic gene expression	[24,44,48]
IGF-1/IGF-2	PI3K/AKT; Ras/Raf/MAPK	PI3K/AKT drives cell survival and ECM synthesis; MAPK improves mitogenic signaling	[53]
TNF-α, IL-6, IL-1β	NF-κB; ERK/MAPK	NF-κB induces inflammatory and fibrotic mediators; ERK promotes migration and MMP production	[59,61]
EGF	ERK/MAPK; PI3K/AKT; JAK/STAT	ERK/MAPK; PI3K/AKT; JAK/STAT stimulates DNA synthesis; supports survival	[62]

**Table 3:** Expression patterns and regulatory mechanisms of extracellular matrix (ECM) components in uterine fibroids compared with myometrium.

ECM Component	Expression Pattern in Fibroid vs. Myometrium	Mechanism Interactions	Role(s) in Fibroid Pathology	GF Regulation	Hormonal Regulation	References
Collagens I and III	High in UFs than MM tissue	Activin A increase mRNA expression through Smad-⅔ signaling	Positive correlation to UF size and cell proliferated markers	TGF-β3, Activin A increase mRNA level in collagens	Collagen biosynthesis was managed by low dose of estrogen	[19,36,40,64]
Proteoglycans	Increased in leiomyoma	Through growth factor signaling	Proteoglycans act as TGF-β antagonists Increase its activation in fibrosis	TGF-β3 stimulates the expression of proteoglycans	Progesterone decreases proteoglycans mRNA expression in fibroid	[19,36,40,64]
Fibronectin	High in estrogen-treated fibroid	PDGF signaling influence production of fibronectin	Cell migration Differentiation Inflammation Bind cells to ECM components	TGF-β3, Activin A increase mRNA level in fibronectin	Estrogen increases fibronectin in UFs	[19,36,40,64]
MMPs (MMP-1, 2, 3, 9)	Expression of MMP-1, -2, -3, -9 is increased in UFs	Prevent apoptosis through Fas/Fas ligand (FasL)	Cell migration Differentiation Inflammation	FGFs, TGF-β VEGF interaction	Estrogen can reduce MMP-2 in leiomyoma	[36,64]
TIMPs (TIMP-1, TIMP-2)	Circulating level of TIMP-1 is high in leiomyoma	MMP activities was regulated by TIMPs	Play essential role in ECM remodeling	Upregulated by TGF-β and steroid hormones	Estradiol activates TIMP-1 expression	[36,64]
Lysyl oxidase (LOX) family	Increased in fibroid tissue	Regulate collagen cross-linking, reduce tumor development, ECM elasticity loss	MMP physiological regulators.	TGF-β together with HIF-1 induce LOX	Estrogen triggers Lox In cervix and vagina	[36,65,66]

## Data Availability

No new data were created or analyzed in this study. Data sharing is not applicable to this article.

## Abbreviations

The following abbreviations are used in this manuscript:

VEGF	Vascular endothelial growth factor
VEGFR-1/2	Vascular Endothelial Growth Factor Receptors 1 and 2
PDGF	Platelet-derived growth factor
PDGF-A/B/C/D	PDGF isoforms
TGF-β	Transforming growth factor-beta
IGF-1/2	Insulin-like growth factor-1/2
EGF	Epidermal growth factor
EGFR	Epidermal growth factor receptor
FGF	Fibroblast growth factor
FGFR	Fibroblast growth factor receptor
MMPs	Matrix metalloproteinases
TIMPs	Tissue inhibitors of metalloproteinases
TNF-α	Tumor necrosis factor-alpha
IL-6, IL-1β	Interleukin-6, Interleukin-1 beta
PLCγ	Phospholipase C-gamma
PKC	Protein kinase C
Raf	Rapidly accelerated fibrosarcoma kinase
MEK	Mitogen-activated protein kinase kinase
MAPK	Mitogen-activated protein kinase
TbRI	Transforming growth factor-β receptor type I
PP2C	Protein phosphatase 2C
mTOR	Mechanistic target of rapamycin
